# Outcomes of Hallux Amputation Versus Partial First Ray Resection in People with Non-Healing Diabetic Foot Ulcers: A Pragmatic Observational Cohort Study

**DOI:** 10.1177/15347346221122859

**Published:** 2022-09-07

**Authors:** Virginie Blanchette, Louis Houde, David G. Armstrong, Brian M. Schmidt

**Affiliations:** 1 Department of Human Kinetics and Podiatric Medicine, 14847 Université du Québec à Trois-Rivières, 3351, boul. des Forges, C.P. 500, Trois-Rivières, Canada, G9A 5H7; 2 Department of Mathematic and Informatic, 14847 Université du Québec à Trois-Rivières, 3351, boul. des Forges, C.P. 500, Trois-Rivières, Canada, G9A 5H7; 3 Southwestern Academic Limb Salvage Alliance (SALSA), Department of Surgery, 12223 Keck School of Medicine of University of Southern California, 1520 San Pablo, St. Los Angeles, CA, 90031, USA; 4 166144 University of Michigan Medical School, Department of Internal Medicine, Division of Metabolism, Endocrinology, and Diabetes, Domino’s Farms (Lobby C, Suite 1300) 24 Frank Lloyd Wright Drive, Ann Arbor, MI, 48106, USA

**Keywords:** diabetes, lower extremity, foot diseases, orthopedic procedures, reoperation, infection

## Abstract

There are few data comparing outcomes after hallux amputation or partial first ray resection after diabetic foot ulcer (DFU). In a similar context, the choice to perform one of these two surgeries is attributable to clinician preference based on experience and characteristics of the patient and the DFU. Therefore, the purpose of this study was to determine the more definitive surgery between hallux amputation and partial first ray resection. We abstracted data from a cohort of 70 patients followed for a 1-year postoperative period to support clinical practice. We also attempted to identify patient characteristics leading to these outcomes. Our results suggested no statistical difference between the type of surgery and outcomes such as recurrence of DFU and amputation at 3, 6, and 12 months or death. However, there was a statistically significantly increased likelihood of re-ulceration for patients with CAD who underwent hallux amputation (p = 0.02). There was also a significantly increased likelihood of re-ulceration for people with depression or a history when the partial ray resection was performed (p = 0.02). Patients with prior amputation showed a higher probability of undergoing another re-amputation with partial ray resection (p = 0.01). Although the trends that emerge from this project are limited to what is observed in this statistical context, where the number of patients included and the number of total observations per outcome were limited, it highlights interesting data for future research to inform clinical decisions to support best practices for the benefit of patients.

Diabetes mellitus (DM) is one of the most common chronic diseases worldwide. DM-related foot complications such as peripheral arterial disease, diabetic foot infection (DFI), diabetic foot ulcer (DFU) and minor or major lower extremity amputation (LEA) reduce the quality of life and lead to premature death.^1,2^ Personal, societal and economic burdens of DFUs highlight the importance to support prevention strategies for the at-risk population as well as effective treatments that will prevent DFU recurrence, re-amputation or other complications such DFI and death.^3,4^ Indeed, DFI is involved in 58% of DFU and approximately 50% of these infected patients are affected with PAD. PAD is highly predictive of LEA.^5–8^ Approximately 17% to 30% of people with a DFU will ultimately require a LEA and patients with DFI have 155 times greater risk of LEA than patients without associated infection.^3,7,9,10^ It is estimated that 85% of all DM-related LEA are preceded by a DFU but sometime, LEAs are an inevitable treatment.^11^

The key components of successful limb salvage are to achieve a DFU-free, plantigrade foot that is functional with treatments that have minimum impact on a patient’s global health. A successful LEA is i) the complete eradication of nonviable tissue to optimize the patient healing potential, ii) reduce the risk of DFU recurrence (or new DFU onset) and iii) avoid the need for extended local wound care or repeat surgical interventions.^12,13^ The goal of isolated partial-foot amputation, such as a hallux amputation and a partial first ray resection, is to maintain bipedal ambulatory status and function.^14,15^ Minor LEA are preferred to major LEA because of their association with less morbidity and mortality.^16,17^ The forefoot has been reported as the most frequent location of DFI in DM.^18^ Furthermore, the metatarsophalangeal joint of the hallux, including sesamoid bones, is more complex from an anatomical perspective than the lesser metatarsophalangeal joints. Such differences in anatomy might impact surgical outcomes.^18^

However, first ray amputations (eg, hallux disarticulation and/or partial first ray amputation) impact a patient’s gait pattern because of the absence of the propulsive phase provided by now altered medial column of the foot.^19,20^ Although those procedures seem to affect gait less than a more proximal LEA, published studies have reported that patients who undergo partial first ray resection often progress to requiring a more proximal repeat LEA.^13,21^ Moreover, following hallux amputation, subsequent higher level of amputation is frequently observed due to new infected DFU associated diabetes limited joint mobility and new ambulatory pattern because of the amputated hallux.^22^

Furthermore, the literature comparing outcomes following hallux amputation or partial first ray resection are limited.^15^ In similar context, the choice to perform one of these two surgeries is attributable to the clinician’s decision according to their experience, to the patient’s DFU characteristics and patient’s preference through informed consent. Hence, guidelines are suggesting clinical decision based on several factors (eg, functional, infection and vascular status, bone quality, presence of infection, etc) with the intent to preserve as much of the limb as possible.^23–28^ The aim was to determine the most definitive surgery between hallux amputation and partial first ray resection for patients with infected ulcer (+ /-) osteomyelitis involving the first ray who were followed for 1-year postoperatively. Our primary objective was to compare DFU events (at the surgical site and/or the ipsilateral foot only) at 3-, 6- and 12-months following the surgical intervention in patients who had hallux amputation or partial first ray resection. Our secondary aim was to compare other outcomes between both cohorts (eg, infection, re-amputation, death). We hypothesized hallux amputation would be most definitive and result in less complications during the 1-year follow up, in line with similar trends from previous studies.^19,21^ It have been reported that patients who undergo partial first ray resection often progress to requiring a more proximal re-amputation.^21^

## Materials and Methods

We performed a observational cohort investigation (retrospective; level of evidence III) which mined and analyzed big data, with coding, from single unified Electronic Medical Records (EMR) at University of Michigan Health System, a large tertiary academic health system overseeing the care of more than 80,000 patients with DM.^29^ Between 2016 and 2020, 70 patients from which 26 had hallux amputation and 44, a partial first ray resection, were retrieved from database and followed for longitudinal outcomes on a one-year period. According to sample size calculation, 38 to 216 patients are sufficient power for confidence interval between 90–95% in the conservative proportion of LEA (17%).^30^ All patients underwent comprehensive medical treatment and surgical intervention by a multidisciplinary team, which included five board-certified podiatric surgeons (for the amputations), nurses, vascular surgeons, and structured and targeted diabetic foot care according to the International Working Group on Diabetic Foot recommendations (IWGDF).^24^

Inclusion criteria were adult DM patients age ≥18 with a concomitant diabetic foot surgery whether hallux amputation or partial first ray resection that EMR reported data over a 1-year period. Our EMR mining system was programmed to include limb salvage procedural codes, based on Common Procedure Terminology (CPT) for higher-level amputations (CPT 84.13-84.19), minor lower extremity amputations (CPT 84.10-84.12). The hallux amputation is defined as the level of amputation distal to the first metatarsophalangeal, including the hallux and the joint.^15,17,31^ Partial first ray resection is defined as the primary amputation of the hallux phalanxes and at least a part of the first metatarsus, distal to the first metatarsal–cuneiform joint and excluded additional digital amputations.^14,17,21^

### Outcomes Measures

Data collected included demographic information (eg, age, sex, race, body mass index, coronary heart disease, hypertension, etc (Table 1). The outcome measures were related DFU healing after the LEA on a 1-year period. DFU healing was defined as a continuous, viable epithelial covering over the entire previously open wound, subsequently within 2 months with no new ulcerations. Complications associated with each surgical approach (DFU at 3-, 6- and 12 months, re-amputation at 3-,6-and 12- months and death) were also collected.

**Table 1.** Baseline characteristics of patients who had partial first ray resection or hallux amputation

Characteristics	Total (n = 70)	Partial First Ray Resection (n = 44)	Hallux amputation (n = 26)	P-value
Age, years, mean ± SD	57.4 ± 11.0	56.3 ± 10.6	59.3 ± 11.2	0.27
Sex, % Men (n)	85.7 (60)	86.4 (38)	84.6 (22)	0.84
Race, % (n) Caucasian Others^†^	78.6 (55) 21.4 (15)	70.5 (31) 29.5 (13)	92.3 (24) 7.7 (2)	0.03*
BMI (kg/m^2^) mean ± SD	32.8 ± 7.0	32.4 ± 7.0	33.4 ± 7.2	0.58
Previous Amputation % (n)	27.1 (19)	27.3 (12)	26.9 (7)	0.22
Presence of SIRS % (n)	11.4 (8)	11.4 (5)	11.5 (3)	0.80
IDSA Classification^25^% (n) 1 : None 2 : Mild 3 : Moderate 4 : Severe	1.4 (1) 41.4 (29) 45.7 (32) 11.4 (8)	0 (0) 36.4 (16) 50 (22) 13.6 (6)	3.8 (1) 50.0 (13) 38.5 (10) 7.7 (2)	> 0.5
Presence of OM^‡^	91.3 (63)	93.2 (41)	88.0 (22)	0.25
CAD % (n)	44.3 (31)	45.5 (20)	42.3 (11)	0.19
HTN % (n)	35.7 (25)	34.1 (15)	38.4 (10)	0.72
CKD stage, % (n) Stage 0 (no CKD) (GFR > 90 mL/min) Stage 1 (GFR = 60–89 mL/min) Stage 2 (GFR = 45–59 mL/min) Stage 3 (GFR = 30–44 mL/min) Stage 4 (GFR = 15–29 mL/min) Stage 5 CKD (GFR <15 mL/min)	57.1 (40) 35.7 (25) 1.4 (1) 2.9 (2) 1.4(1) 1.4 (1)	63.6 (28) 27.3 (12) 0 (0) 2.5 (2) 2.3 (1) 2.3 (1)	46.2 (12) 50.0 (13) 3.8 (1) 0 (0) 0 (0) 0 (0)	> 0.5
Smoking, % (n)	32.9 (23)	31.8 (14)	34.6 (9)	0.81
DPN	91.4 (64)	93.2 (41)	88.5 (23)	0.50
PLT, K/uL, mean ± SD	267.8 ± 118.4	277.5 ± 133.9	251.4 ± 86.4	0.52
HBG g/dL, mean ± SD	11.7 ± 1.8	11.4 ± 2.0	12.1 ± 1.3	0.02*
ESR mm/hr, mean ± SD^#^	73.6 ± 36.3	82.35 ± 37.3	58.9 ± 27.7	0.04*
MCV fI, mean ± SD	86.4 ± 6.6	85.6 ± 6.4	87.7 ± 6.9	0.82
Glucose mg/dL, mean ± SD^¶^	206.5 ± 125.1	228.1 ± 136.4	170.9 ± 95.6	0.08
C-RP mg/dL, mean ± SD^¥^	11.5 ± 9.7	13.09 ± 9.5	8.65 ± 9.6	0.98
TBI, mean ratio (amputation side when possible)^§^	0.55 ± 0.24	0.51 ± 0.25	0.63 ± 0.17	0.12
Non compressible vessel due to calcification^§^	22.2 (12)	22.9 (8)	22.2 (4)	0.99
Previous revascularization, % (n)	11.4 (8)	15.0 (6)	7.7 (2)	0.45
Depression^&^ % (n)	15.7 (11)	15.9 (7)	15.4 (4)	0.95
Ulcer classification (UT)^35^ previous to amputation, % (n) 1B 2B 2C 2D 3A 3B 3C 3D 4B 4D Missing data	2.9 (2) 17.1 (12) 1.9 (1) 7.1 (5) 4.3 (3) 27.1 (19) 1.9 (1) 8.6 (6) 12.9 (9) 4.3 (3) 12.9 (9)	2.3 (1) 9.1 (4) 0 (0) 11.4 (5) 2.3 (1) 27.3 (12) 0 (0) 11.4 (5) 18.1 (8) 4.5 (2) 13.6 (6)	3.8 (1) 30.8 (8) 3.8 (1) 0 (0) 7.7 (2) 26.9 (7) 3.8 (1) 3.8 (1) 3.8 (1) 3.8 (1) 11.5 (3)	> 0.05

Legend:

^†^ Black or Asian people

^‡^ Calculated with n = 69 (1 missing datum for the hallux amputation group)

* Statistically significant

^#^ Calculated with n = 62 (2 missing data for hallux amputation group and 4 missing data for partial ray resection group).

^¶^ Calculated with n = 69 (1 missing datum for the partial ray resection group)

^¥^ Calculated with n = 66 (2 missing data for hallux amputation group and 4 for the partial ray resection group)

^§^ Calculated with n = 14 for hallux amputation group (7 missing data and 4 calcification values) and n = 27 for partial ray resection group (9 missing data and 8 calcification values)

^&^ Depression was diagnosed by the team using the DMS-V criteria, a tool and reference guide for mental health clinicians to diagnose, classify, and identify mental health conditions.

Abbreviations:

SD: Standard Deviation; BMI: Body Mass Index; OM: Osteomyelitis confirmed by radiograph or Magnetic Resonance Imagery; CAD: Coronary Arterial Disease; HTN: Hypertension; SIRS: Systemic Inflammatory Response Syndrome; CKD: Chronic Kidney Disease; GFR: Glomerular Filtration Rate; DPN: Diabetic peripheral neuropathy suspected by loss of protective sensation and clinical findings; MCV: Mean corpuscular volume; HBG: hemoglobin; ESR: erythrocytes sedimentation rate; PLT: Platelet level; CR-P: C-Reactive Protein Level; TBI: Toe-Brachial Index

### Data Analysis

Demographic data were analyzed using descriptive statistics. To compare the grades both groups, the characteristics were analyzed using chi square (*χ*2). Re-ulceration and re-amputation (or better ulcer-free and amputation-free survival) are time-dependent measures that can be reported as Kaplan-Meier curves. However, our retrospective data have allowed only time estimates (in months; not precise, as they were agglomerated). Since we cannot be very precise related to the time, which is important in Kaplan-Meier curves, we performed Mann-Whitney U test (non-parametric) and Friedman test on the independent samples to compare the means of the quantitative variables related to the outcomes. When the sample sizes were not sufficient to the accurate p-value we did adjustment using a bootstrap method. We performed a multivariate logistic regression per variable for patients’ characteristics known to be predictor factors for DFU and LEA according to the literature and our previous work.^32^ Odd ratio was the association measure for continuous data. The *χ*2 was used to measure the independence of the dichotomous and multinomials variables between surgical type (hallux amputation or partial ray resection), the outcomes (cumulative re-ulceration or re-amputation) related to the variable interest. Odd ratios cannot be calculated in this statistical context. This was expressed using proportion. The death as outcomes could not be assessed with the regression because there were too few events for the sample size. P-value inferior to 0.05 was considered a significant association between outcomes and those factors in this analysis. This study is reported according to the STROCSS 2019 guidelines.^33^ It was approved by the Institutional Review Board (HUM00108607) and it was completed in accordance with the ethical standards of the Ethics Committee. We used SPSS Statistics software 27 (IBM Corp, New York, United States) to perform the analysis.

## Results

### Demographics and Clinical Characteristics

A total of 70 patients who underwent first ray amputation surgery or hallux amputation were included in the study. The total cohort is mainly Caucasian (78.8%) male (85.7%) with an average age of 57.4 years (Table 1). DFU clinical presentation during hospital admission was primarily used to determine necessity of operative intervention. Ten patients (38.4%) in the hallux cohort and fifteen patients (34.1%) in the partial first ray cohort had index DFU on the left foot requiring surgical intervention. Neuropathic wound etiologies accounted for 92.2% and 88.5% in the hallux and partial first ray amputation cohorts, respectively. Although we had missing data for the vascular component, calcified vessels accounted for 22.2% and limited accurate reporting of vascular status. It is known at least 11.4% of the cohort had prior revascularization and ischemia was mild to moderate.^34^ However, the majority of DFU were classified according to the University of Texas classification which accounts for an ischemic component of the index DFU (ie, class C or D).^35^ All patients except one (in the partial ray resection cohort) were ambulatory prior to the amputation.

Pre-operative imaging was obtained in all patients to assist in operative planning. Radiographs were obtained in all patients and advanced imaging via magnetic resonance imaging (MRI) was obtained in 25 (56.8%) and 18 (69.2%) in the partial first ray and hallux amputations cohorts, respectively (p > 0.05). The rates of OM diagnosed was (93.2 .0% v. 88.0%, p = 0.25). Prior to amputation, Charlson Comorbidity Index (CCI) values (5.4 ± 3.5 v. 4.7 ± 2.6; p > 0.05), IDSA classification at time of admission (2.5 ± 0.7 v. 2.8 ± 0.7; p > 0.32), leukocyte count (9.4 ± 4.6 v. 12 ± 6.9; p > 0.05) were similar. Patient characteristics were relatively similar and did not reach statistical significance (p > 0.05) for all variables (Table 1). Inflammatory markers including erythrocyte sedimentation rate (ESR) and C-reactive protein (C-RP) demonstrated divergence in our population. ESR demonstrated increased elevation in the partial ray group versus the hallux cohort (58.9 ± 27.7 v. 82.4 ± 37.3 p = 0.04), but the acute phase reactant C-RP did not demonstrate a difference (8.6 ± 9.6 v. 13.1 ± 9.5; p = 0.98). The partial first ray resection group was more ethnically diverse (29.5% v. 7.2%; p = 0.03) and also had a lower hemoglobin level (11.4 ± 2.0 v. 12.1 ± 1,3; p = 0.02).

### Outcomes

Of the 70 patients, all had defined primary outcomes at 1 year (Table 2). In the hallux amputation group, six (23%), three (12%), and two (8%) developed ulcer recurrence within 3-, 6-, and 12 months post-operatively, respectively. Similarly, in the partial ray group, 16 (36%), 8 (18%), and 6 (14%) developed re-ulceration within 3-, 6-, and 12-months postoperative follow-up, respectively. The difference among cohorts did not reach statistical significance. Re-amputation occurred in 0, 3 (12%), and 0 patients and 6(14%), 5(11%), and 1 (2%) in the hallux and partial first ray amputation groups, respectively, at 3-, 6-, and 12-months follow-up periods. The difference in rate of re-amputation was not significant at any time point in longitudinal follow-up. Additionally, two patients in the hallux amputation group and four in the partial first ray amputation group died; no deaths were related to surgical intervention or foot infection.

**Table 2.** Primary outcomes comparison

	3-month ulcer n (%)	3-month amputation n (%)	6-month ulcer n (%)	6-month amputation n (%)	12-month ulcer n (%)	12-month amputation n (%)	Death^†^ n (%)
**Partial First Ray Resection (n = 44)**	16 (36)	6 (14)	8 (18)	5 (11)	6 (14)	1 (2)	4 (9)
**Hallux Amputation (n = 26)**	6 (23)	0 (0)	3 (12)	3 (12)	2 (8)	0 (0)	2 (8)
**p-value**	0.295	0.078	0.521	1	0.701	1	1

Legend :

^†^ During the year following the indexed amputation surgery.

### Factors Associated with Outcomes and Surgical Procedures

Although the association was not statically significant for chronic kidney disease (CKD) (Table 3), a trend was observed in the association between having a re-ulceration at one-year and having undergone partial ray resection amputation versus hallux amputation (OR 4.15 vs 0.53; p > 0.05). In terms of baseline demographic, clinical and laboratory characteristics, only three factors were found to influence outcomes with statistically significant differences (Table 4). Patients had a higher probability of re-ulceration in the hallux amputation cohort (54.5%; p = 0.02) if they had coronary artery disease (CAD). The same was not true in the partial ray resection cohort (45.0%; p = 0.96). For patients with current or a history of depression (not specified in the EMR), the partial first ray resection cohort had more re-ulcerations (85.7%; p = 0.02) compared with the likelihood of the hallux cohort (50%; p = 0.37). A higher probability to have a re-amputation was found for patient in the partial ray resection cohort (58.3%; p = 0.01) compared to the other cohort probability (14.3%; p = 0.79) when they presented with a prior history of amputation.

**Table 3.** Logistic regression for continuous data: relationship between surgical type, the outcomes (re-ulceration or re-amputation) and related to the variable.

Variable	Surgery Types	OR	P-Value
Total ulceration (3-, 6- and 12-months)			
Age	HA	0.98	0.46
	PRR	1.01	0.57
BMI	HA	1.08	0.12
	PRR	1.16	0.44
PLT	HA	1.00	0.91
	PRR	0.99	0.25
HBG	HA	1.26	0.17
	PRR	1.27	0.98
MCV	HA	1.07	0.17
	PRR	1.04	0.67
TBI^†^	HA	0.75	0.79
	PRR	0.71	0.97
Glucose	HA	1.00	0.57
	PRR	1.00	0.54
ESR	HA	0.99	0.27
	PRR	1.04	0.54
C-RP	HA	0.95	0.18
	PRR	1.00	0.44
CKD	HA	0.53	0.13
	PRR	4.15	0.11
Re-amputation (3-, 6- and 12-months)			
Age	HA	1.05	0.19
	PRR	1.05	0.98
BMI	HA	0.99	0.87
	PRR	1.13	0.26
PLT	HA	1.00	0.74
	PRR	0.84	0.97
HBG	HA	1.03	0.87
	PRR	0.94	0.85
MCV	HA	1.07	0.23
	PRR	1.17	0.52
TBI	HA	0.32	0.37
	PRR	0.52	0.86
Glucose	HA	1.00	0.50
	PRR	1.00	0.94
ESR	HA	1.02	0.13
	PRR	0.97	0.09
CRP	HA	0.98	0.62
	PRR	1.01	0.65
CKD	HA	1.25	0.43
	PRR	1.09	0.94

Legend:

^†^ Calculated with n = 13 for hallux amputation group (8 missing data) and n = 27 for partial ray resection group (9 missing data), excluding 13 patients with non-compressible vessel due to calcification (see table 1)

Abbreviations:

OR: Odd Ratio; HA: Hallux amputation; PRR: Partial Ray Resection; BMI: Body Mass Index; PLT: Platelet level; HBG: hemoglobin; MCV: Mean corpuscular volume; TBI: Toe-Brachial Index; ESR: Erythrocytes Sedimentation Rate; CR-P: C-Reactive Protein Level

**Table 4.** Probability of re-ulceration or re-amputation with dichotomous and multinomial variables by amputation type

Variable	Surgery types	Proportion, %	P-Value
Total ulceration (3-, 6- and 12-months)			
Sex (men)	HR	27.3	0.37
	PRR	42.1	0.26
Race (Caucasian)	HR	33.3	0.33
	PRR	50.0	0.43
Previous amputation	HR	42.9	0.42
	PRR	58.3	0.29
SIRS	HR	0	0.22
	PRR	50.0	0.81
OM	HR	27.3	0.17
	PRR	46.3	0.66
IDSA 1; 2; 3; 4	HR	0; 7.8; 50.0; 50.0	0.26
	PRR	0; 56.3; 40.9; 33.3	0.52
CAD	HR	54.5	0.02*
	PRR	45.0	0.96
HTN	HR	16.7	0.39
	PRR	42.9	0.74
Smokers	HR	55.6	0.46
	PRR	42.9	0.81
DPN	HR	30.4	0.92
	PRR	46.6	0.66
Previous vascularization	HR	0	0.33
	PRR	50.0	0.81
Depression	HR	50.0	0.37
	PRR	85.7	0.02*
Re-amputation (3-, 6- and 12-months)			
Sex (men)	HR	13.6	0.43
	PRR	23.7	0.18
Race (Caucasian)	HR	12.5	0.60
	PRR	25.0	0.47
Previous amputation	HR	14.3	0.79
	PRR	58.3	0.01*
SIRS	HR	0.0	0.51
	PRR	50.0	0.18
OM	HR	9.1	0.23
	PRR	29.3	0.27
IDSA^25^ 1; 2; 3; 4	HR	0; 7.7; 20; 0	0.73
	PRR	0; 25.0; 27.3; 33.3	0.93
CAD	HR	18.2	0.36
	PRR	35.0	0.29
HTN	HR	0	0.31
	PRR	14.3	0.24
Smokers	HR	22.2	0.22
	PRR	28.6	0.90
DPN	HR	13.0	0.51
	PRR	29.3	0.27
Previous vascularization	HR	0	0.60
	PRR	33.3	0.72
Depression	HR	25.0	0.36
	PRR	28.6	0.93

Legend:

* Statistically significant

Abbreviations:

SIRS: Systemic Inflammatory Response Syndrome; OM: Osteomyelitis; IDSA: Infection Disease Society of America Classification; CAD: Coronary Arterial Disease; HTN: Hypertension; DPN: Diabetic Peripheric Neuropathy; SIRS: Systemic Inflammatory Response Syndrome.

## Discussion

This study reported outcome difference between hallux amputation and partial first ray resection in a retrospective patient cohort of 70 patients followed on 1-year postoperative period and intended to support decision-making. Although the groups were slightly different at the baseline, especially related to two laboratory tests (HBG and ESR), the characteristics of the DFU, age and sex were similar. HBG and ESR, respectively associated with anemia and infection, are recognized as markers of morbidity and mortality in patients with DFU and to increase amputation risk.^36,37^ Moreover, there was a greater population’s diversity in the partial ray resection cohort, which could have also influenced the results. Indeed, it is well known that some ethnicity undergo more major amputations.^38^ Recent studies have demonstrated that American Africans have more minor LEA when they have DFU infection, but there is less LEA in the Asian population.^39,40^ As a result, we would have expected to observe more outcomes in the partial ray resection group. However, our results did not show significant differences related to re-ulceration, re-amputation, or death. Thus, our results are partially in agreement with those of a previous study specifically on partial first ray amputation, which reported this type of surgery often progresses to a more proximal LEA and increases the risk of DFU.^19^

In the context of this study, we identified factors such as depression and CAD are associated with more re-ulceration depending on the type of surgery. Patient with previous amputation was also associated with more re-amputation in the partial ray resection group which is consistent with previous study.^21^ Moreover, depression was also highlighted as a predictor to LEA.^41^ While not statistically significant, a partial ray first resection with CKD can lead to more re-ulceration compared to the hallux amputation (OR 4.15 vs 0.53). Our results are again consistent with a previous systematic review.^42^ Therefore, these findings suggest a partial first ray resection should be avoided in patients with the following characteristics: CKD, depression or a history, and a previous amputation. It may support, to some extent when the presentation of the infection permits, the clinical decision to avoid this surgical procedure to reduce the likelihood of a poor (future) prognosis.

Overall, approximately 59% of patients had a re-ulceration and 21% had a re-amputation within one year in our cohort. In parallel with earlier literature reports which demonstrate approximately 60% of patients will need further LEA and 46% will have an DFU recurrence.^13,43,44^ However, the mortality rate of approximately 9% was lower than the one reported in a recent systematic review (approximately 20%).^42^ This positive finding can be justified by the diabetic foot management at our institution including a specialized service with a team approach to diabetic foot disease including podiatry.^45^ This approach has been recognized to improve diabetic foot outcomes and enhance quality of care.^46,47^ Although this is a hypothesis, the lower mortality rate should be further explored, particularly as the data from this project did not allow for differentiation of major and minor LEA as outcomes. It is recognized that mortality and poor quality of life are higher in DM patients who undergo major LEAs.^3^ This type of data would have been informative and represents a limitation.

There are also other limitations to this study. First, this is an observational study; therefore, there is no control group and some missing data (Table 1). Second, providers chose surgical intervention based on clinical appearance and radiographic findings. There was no structured algorithm to guide surgeons in their decision-making, and thus the dataset was dependent on standard of care as described by IWGDF. However, there were only five board-certified surgeons involved and reduced bias in decision-making and limited excessive heterogeneity. In fact, the design of the study is pragmatic in that it aims to answer a practical clinical question to support decision-making and potentially is helpful to guide therapy.

More specific continuous measurement variables, such as albumin and (absolute) toe pressures, were not available for comparison and a better understanding of the vascular and healing potential are essential. However, these are not routinely performed in inpatient assessment at our institution. In addition, analysis was complicated by missing data but also because of the low number of events at each time of follow-up. Additional information on these variables collected at uniform timelines could provide improved granularity into optimal procedure selection for a given patient. The statistical context limits the generalizability of the results. However, further prospective study in this area could also inform, in addition to health outcomes, about benefit, harms, adverse events and satisfaction or other patient-related outcomes to better support shared-clinical decisions (between patients and providers) in DFI context. This study highlighted future hypotheses exploration such as whether the complication rates of hallux amputations are worse first in a particular population (ie, with CAD or other comorbidities /risk factors), and thus whether these individuals should have a partial first ray amputation at the first place to achieve the best outcome.

To date, the decision to perform a partial first ray amputation or hallux amputation (disarticulation) was based on provider decision-making and not evidence-based medicine with respect to outcomes. Our cohort, although small (n = 70), demonstrates no significant difference in patient outcomes at one-year following surgical intervention. This included outcomes such as re-ulceration, re-amputation, and death. When faced with an infected ulcer ( + /-) osteomyelitis involving the first ray, if the infection can be eradicated through the removal of additional bone (partial first ray instead of hallux amputation), this decision is supported by evidence to be as safe as a hallux disarticulation without additional long-term sequelae of the operation from this study. However, consideration should be given when the patient outlined characteristics identified by this study. From an overall perspective, lower mortality at 1-year of our cohort supports the importance of team management of this health issue.

## Conclusion

This study highlights interesting data to inform clinical decisions to support best practices for the benefit of patients with respect to osteomyelitis in the first ray. Future research should guide surgeons in their decision-making to incorporate evidence-based medicine approaches to diabetic foot infections *before* intervention rather than to continue to operate blindly with respect to eventual clinical outcomes.
